# Pseudomonas aeruginosa keratitis misdiagnosed as fungal keratitis by in vivo confocal microscopy: a case report

**DOI:** 10.1186/1756-0500-7-907

**Published:** 2014-12-13

**Authors:** Jiaxu Hong, Qihua Le, Sophie X Deng, Wenjun Cao, Jianjiang Xu

**Affiliations:** Department of Ophthalmology and Visual Science, Eye, Ear, Nose, and Throat Hospital, School of Shanghai Medicine, Fudan University, 83 Fenyang Road, Shanghai, 200031 China; Cornea Division, Jules Stein Eye Institute, University of California, Los Angeles, CA 90095 USA; School of Life Sciences, Fujian Provincial Key Laboratory of Ophthalmology and Visual Science, Xiamen University, Xiamen, 361005 China; Schepens Eye Research Institute, Harvard Medical School, Boston, Massachusetts 02114 USA

**Keywords:** *Pseudomonas* aeruginosa keratitis, Fungal keratitis, *In vivo* confocal microscopy

## Abstract

**Background:**

To report a case of non-typical *Pseudomonas* aeruginosa keratitis that was misdiagnosed as fungal keratitis by *in vivo* confocal microscopy.

**Case presentation:**

A 37-year-old Chinese woman presented with a 2-week history of increasing pain and redness of the right eye. She was started on hourly topical fortified tobramycin and levofloxacin by the referring doctor without improvement. She denied any improvement of her symptoms and signs. On examination, she had a large central corneal ulcer extending to the peripheral cornea. Further symptoms included a satellite lesion, intense conjunctival injection and marked corneal oedema. The corneal scrape was not performed initially because of the deep infiltrate in the stroma. The patient was examined by *in vivo* confocal microscopy. Confocal microscopy images showed hyper-reflective, thin, and branching interlocking linear structures in the stroma that were 5–8 μm in width and 200–400 μm in length. The morphology was consistent with that of fungus. However, the histopathological examination, Gram stain, and culture of the cornea only confirmed the presence of a Pseudomonas species within the deep strom. No fungal element was found. The pathogen was sensitive to ciprofloxacin, gentamicin, levofloxacin, tobramycin and amikacin.

**Conclusion:**

This case reports the potential for a false positive finding of fungus in *Pseudomonas* aeruginosa keratitis and emphasizes the importance of bacterial culture and antibiotic susceptibility testing in the management of microbial keratitis.

## Background

In vivo confocal microscopy (IVCM) enables microstructural analysis of the cornea. Multiple articles reported that amoebic, bacterial, and fungal organisms were detected in vivo in infectious keratitis [1]. Pseudomonas aeruginosa keratitis (PAK) usually progresses rapidly and presents with suppurative stromal infiltrate and marked mucopurulent exudate. However, there are few articles reporting the IVCM finding of PAK [2]. In this report, we present a case of non-typical PAK that was misdiagnosed as fungal keratitis by IVCM.

## Case presentation

A 37-year-old Chinese woman presented with a 2-week history of increasing pain and redness of the right eye. She denied any history of ocular trauma or contact lens wear. Her ocular history included bilateral high myopia and retinal detachment in the left eye in twelve years prior. She had no known drug allergies and no systemic infections at the time of her presentation. She was started on hourly topical fortified tobramycin and levofloxacin by the referring doctor without improvement. She denied any improvement of her symptoms and signs. A septic screening, including a chest x-ray and blood cultures, was negative.

**Figure 1:**
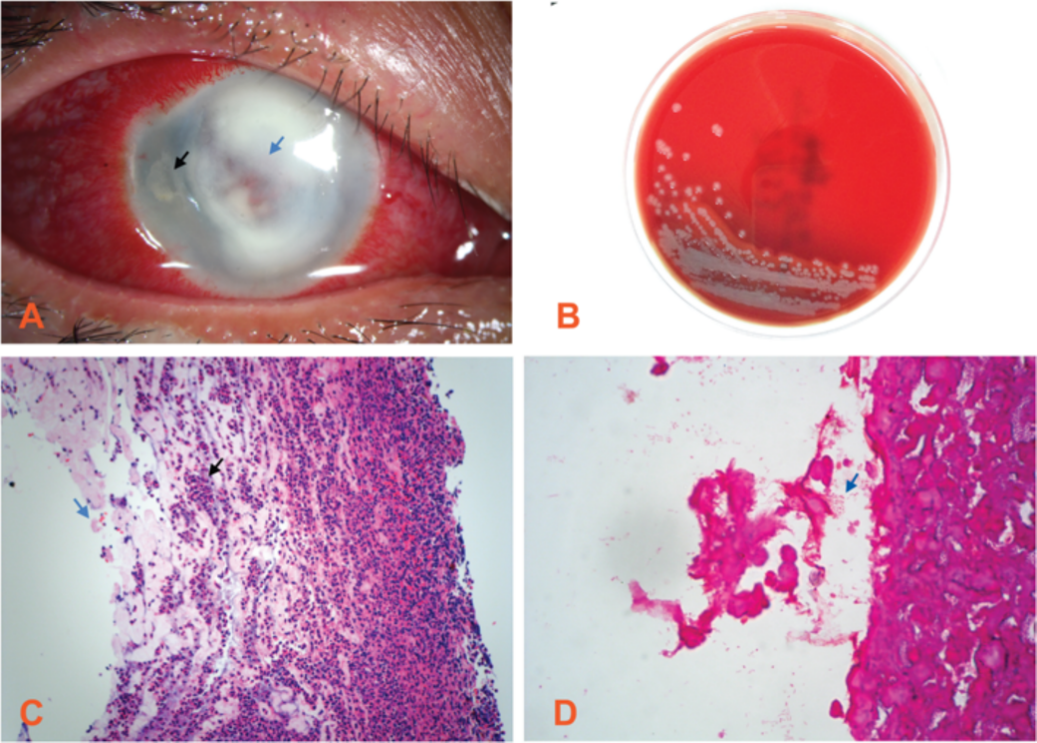
**A** ***Pseudomonas*** **aeruginosa keratitis case. (A)** Slit lamp microscopic image of severe central corneal infiltrate (blue arrow) with intensive conjunctival injection and a temporal satellite lesion (black arrow). Magnification: ×10. **(B)** Microbiological cultures obtained from a superficial corneal swab showed the presence of *Pseudomonas* aeruginosa. **(C)** Hematoxylin and eosin stains demonstrate that the corneal specimen contains numerous polymorphonuclear leukocytes (black arrow) and the epithelium and endothelium are absent (blue arrow). The lamellar architecture is lost and the frayed collagen is the result of widespread collagenolysis. Magnification: ×40. **(D)** Gram staining shows that *Pseudomonas* species could be found in the corneal deep stroma, which appear as short stubby rods and are Gram negative (blue arrow). Magnification: ×100.

**Figure 2:**
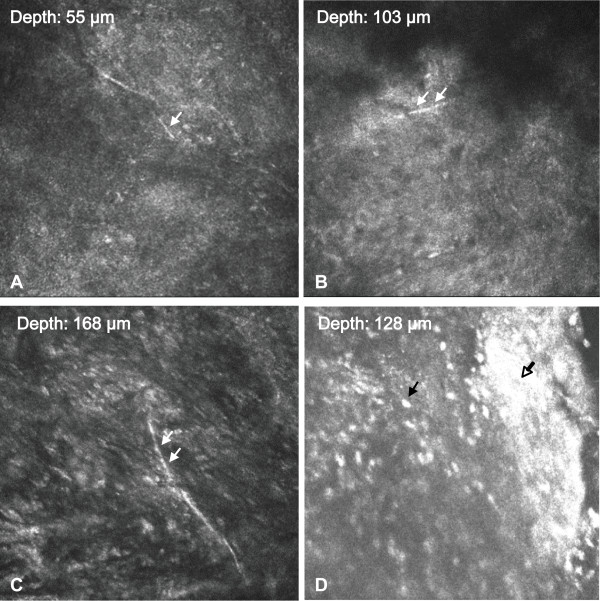
**In vivo confocal microscopy examination. (A~C)** Images from different depth show hyper-reflective branching hyphae-like bodies (white arrow) could be identified in the cornea. **(D)** Infiltration of inflammatory cells (black arrow) and necrotic tissues (hollow arrow). Magnification: ×800.

On examination, her best-corrected visual acuities were light perception in the right and non-light perception in the left. She had a large central corneal ulcer (Figure 1A) extending to the peripheral cornea. A satellite lesion, intense conjunctival injection, and marked corneal oedema were present. The corneal scrape was not performed initially because of the deep infiltrate in the stroma. The patient was examined with an IVCM (Heidelberg Engineering, Heidelberg, Germany). Interestingly, IVCM images showed hyper-reflective, thin, and branching interlocking linear structures in the stroma that were 5–8 μm in width and 200–400 μm in length. The morphology was consistent with that of fungus by other articles (Figure 2A and B). These hyper-reflective structures were surrounded by infiltration of inflammatory cells (Figure 2C and D). Topical amphotericin B, natamycin, and systemic itraconazole were initiated immediately. A penetrating keratoplasty was performed after perforation occurred 3 days later. However, the histopathological examination, Gram stain, and culture of the cornea only confirmed the presence of a *Pseudomonas* species deep within the stroma (Figure 1B~D). No fungal element was found. The pathogen was sensitive to ciprofloxacin, gentamicin, levofloxacin, tobramycin and amikacin.

## Discussion

Diagnosis of PAK can be challenging if patients show the development of satellite lesions which are often cited as a hallmark of fungal keratitis [3]. Although IVCM has been used more frequently to diagnose fungal keratitis, false positive results have been reported. Shi *et al.* first reported that IVCM may not be useful in all patients with fungal keratitis, especially at the late stage when a low number of hyphae are difficult to identify [4]. Hau *et al.* also found that *Nocardia* and other bacterial keratitis could be misdiagnosed as fungal keratitis by IVCM, because linear hyphae-like opacities can be easily confused with fungal hyphae [5]. In addition, Vaddavalli *et al.* demonstrated that the subjects of 4 of 45 bacterial keratitis cases were misdiagnosed as having fungal filaments on confocal microscopy [6]. Notably, although the *in vitro* culture results indicated that the *Pseudomonas* species in this case was sensitive to levofloxacin and tobramycin, the patients seemed to be initially unresponsive to hourly topically-fortified tobramycin and levofloxacin eye drops. A possible explanation is that most of the pathogen was located in the deep stroma which may make it hard for the drugs to reach an effective concentration. In addition, because *Pseudomonas aeruginosa* and fungal keratitis are not exclusive of each other, and the patient first received anti-fungal treatment followed by antibiotic treatment, it seemed that the co-infection was possible. However, no fungus was identified in the microbiological smear, culture, or on histological examinations.

Thus far, no articles have reported comparisons of the differences among IVCM findings between fungal and pseudomonas keratitis. It has been reported that the diagnostic accuracy of microbial keratitis by confocal microscopy is mainly dependent on observer experience [5]. Difficulty in distinguishing host cells from pathogenic organisms limits the value of IVCM as a stand-alone tool in differentiating different kinds of microbial keratitis. The common criterion used for the identification of fungal filaments by IVCM was the presence of highly reflective filaments varying in size between 3 and 8 μm. These filaments were of uniform width with an irregular branching pattern and were not seen in isolation. Other linear structures that could be confused with fungal filaments included nerve fibers, collagen fibrils, scar tissue, blood vessels, and striae [6].

## Conclusion

To our knowledge, this is the first report of PAK that was misdiagnosed as fungal keratitis by IVCM. This case reports the potential for false positive findings of fungus in PAK and emphasizes the importance of bacterial culture and antibiotic susceptibility testing in the management of microbial keratitis.

## Consent

Written informed consent was obtained from the patient for publication of this case report and any accompanying images. A copy of the written consent is available for review by the editor-in-chief of this journal.

## Abbreviations

IVCM: *In vivo* confocal microscopy
PAK: Pseudomonas aeruginosa keratitis.
